# Excellent capability for molecular detection of *Aedes*-borne dengue, Zika, and chikungunya viruses but with a need for increased capacity for yellow fever and Japanese encephalitis viruses: an external quality assessment in 36 European laboratories

**DOI:** 10.1128/jcm.00910-24

**Published:** 2024-12-16

**Authors:** Lance D. Presser, Cécile Baronti, Ramona Moegling, Laura Pezzi, Yaniv Lustig, Céline M. Gossner, Chantal B. E. M. Reusken, Rémi N. Charrel, on behalf of EVD-LabNet

**Affiliations:** 1 National Institute for Public Health and the Environment (RIVM), Center for Infectious Disease Control 571105 , Bilthoven, the Netherlands; 2 Unite des Virus Emergents (UVE: Aix-Marseille Univ, Universita di Corsica, IRD 190, Inserm 1207, IRBA), Marseille, France; 3 National Reference Center for Arboviruses, Inserm-IRBA, Marseille, France; 4 Central Virology Laboratory, Public Health Services, Ministry of Health and Sheba Medical Center, Ramat-Gan, Israel; 5 Disease Programme Unit, European Centre for Disease Prevention and Control 56756 , Solna, Sweden; 6 Laboratoire des Infections Virales Aigues et Tropicales, AP-HM Hôpitaux Universitaires de Marseille 36900 , Marseille, France; Mayo Clinic Minnesota, Rochester, Minnesota, USA

**Keywords:** external quality assessment, EQA, chikungunya virus, zika virus, dengue virus, yellow fever virus, Japanese encephalitis virus, arbovirus, orthoflavivirus, alphavirus

## Abstract

Mosquito-borne viruses represent a large global health burden. With geographic expansion of competent vectors for chikungunya virus (CHIKV), dengue virus (DENV), and Zika virus (ZIKV) in Europe, it is anticipated that the number of autochthonous cases of these tropical viruses in Europe will increase. Therefore, regular assessment of diagnostic capabilities in Europe is important. Our aim was to evaluate the mosquito-borne virus molecular detection capability of expert European laboratories by conducting an external quality assessment in October 2023. Molecular panels included 12 plasma samples: one alphavirus (CHIKV), four orthoflaviviruses (ZIKV, yellow fever virus [YFV], DENV, and Japanese encephalitis virus [JEV]), and two negative control samples. Mosquito-borne virus detection was assessed among 36 laboratories in 24 European countries. Adequate capabilities were lacking for YFV and JEV. Many laboratories relied on a mix of laboratory-developed tests (some of which were pan-orthoflavivirus or pan-alphavirus in combination with sequencing) and commercial assays. 47.2% of laboratories characterized all external quality assessment (EQA) samples correctly. Correct result rates were 100% for CHIKV and ZIKV and >99% for DENV, but laboratories lacked capacity, specificity, and sensitivity for JEV and YFV. Three of the viruses in this panel emerged and transiently circulated in Europe: CHIKV, ZIKV, and DENV. Molecular detection was excellent for those viruses, but <50% is accurate for the remainder of the panel. With the possibility or continuation of imported cases and a growing global concern about climate change and vector expansion, progress toward rapid, accurate mosquito-borne virus diagnostics in Europe is recommended, as well as regular EQAs to monitor it.

**IMPORTANCE:**

The external quality assessment (EQA) focused on *Aedes*-borne viruses: chikungunya virus (CHIKV), dengue virus (DENV), Zika virus (ZIKV), and yellow fever virus (YFV). Japanese encephalitis virus, an orthoflavivirus that is spread by mosquito species belonging to the genus *Culex*, was included in the quality assessment as well. CHIKV, DENV, and ZIKV have proven potential for transient and limited circulation in Europe upon introduction of viremic travelers returning to *Aedes albopictus*-endemic regions. Results of this EQA were excellent for those viruses, but <50% is accurate for the remainder of the panel (YFV and Japanese encephalitis virus). Considering imported cases and the threat of climate change and competent vector expansion, progress toward rapid, accurate mosquito-borne virus diagnostics in Europe is recommended.

## INTRODUCTION

Arthropod-borne viruses exert an increasing pressure on health globally (1–7). The World Health Organization (WHO) estimates the yearly global burden for dengue virus (DENV) at 96 million clinical cases including 400,000 deaths while four billion people are at risk for infection around the world (8). It has been predicted that an additional 2.25 billion people (range 1.27–2.8) will be at risk for infection by 2080 (4).

In the past few decades, viruses transmitted by the mosquito species *Aedes albopictus* and *Aedes aegypti* have increasingly spread across the world causing significant outbreaks in naïve populations (9, 10). Such propensity for dissemination of *Aedes*-borne viruses was exemplified in 2013–2014 when chikungunya virus (CHIKV) and in 2015–2016 when Zika virus (ZIKV) entered and spread in the Americas (11, 12). Currently (2023–2024), a global upsurge in DENV infections with >5 million cases including ~5000 deaths across the world is being reported to WHO, including reports from the European region (13) .

Local transmission of originally (sub)tropical viruses like CHIKV, DENV, and ZIKV has been reported in Europe since 2007 from areas with established *Ae. albopictus* populations (14). Local transmission of DENV has been reported from Croatia, France, Italy, Portugal, and Spain, with two cases of dengue related to travel within Europe reported in 2020 (the Netherlands ex-France) (15–20). In 2023, a record number of 130 autochthonous DENV cases were reported to the European Centre for Disease Prevention and Control (ECDC), with cases as far north as Paris, France (16). Local transmission of CHIKV has been reported from France and Italy with outbreaks of hundreds of cases in Italy in 2007 and 2017 while a few local mosquito-borne ZIKV cases were reported by France in 2019 (21–24). While these three viruses have no established endemic transmission cycle in Europe, the risk of transient local transmission is directly linked to the risk of virus importation through viremic travelers returning from *Ae. albopictus*- and *Ae. aegypti*-infested areas (14, 25). With the increasing health burden of *Aedes*-borne virus circulation worldwide (6, 7), the risk for virus importation through returning travelers increases as well (26–29). It is predicted that ongoing climate change will make Northern European regions increasingly suitable for *Ae. albopictus* establishment, which parallels an expansion of at-risk areas for autochthonous transmission of human-to-human transmissible viruses vectored by this mosquito species (30). *Ae. albopictus* and *Ae. aegypti* are considered invasive mosquito species, and introductions (but no establishment yet) have been described as far north as Sweden for *Ae. albopictus* and the Netherlands for *Ae. aegypti* (14, 25).

Besides these three viruses, other *Aedes*-borne viruses with a potential significant health burden, like yellow fever virus (YFV), are sporadically introduced in Europe through returning travelers (29, 31, 32). While YFV epidemics elsewhere in the world can be linked to an increase of imported cases to Europe, these introductions have not yet resulted in autochthonous transmission in *Ae. albopictus-* or *Ae. Aegypti*-endemic areas.

Therefore, it is no wonder that *Aedes*-borne viruses that threaten human health are increasingly the focus of (inter) national preparedness, readiness, and response activities (33, 34). One of the main components of outbreak and pandemic preparedness and control is the ability to diagnose infections timely and accurately (35–37). For this and for the purpose of reliable surveillance for EU-notifiable arboviral diseases, laboratory functions in European Union and European Economic Area (EU/EEA) countries need an adequate capacity and capability to detect. Therefore, there is a need for periodic assessment of the capability of laboratories for detection of emerging arboviruses that represent a public health threat. To strengthen the molecular diagnostic capability in the EU/EEA and EU-enlargement countries for emerging arboviruses, an external quality assessment (EQA) through proficiency testing was performed among members of the ECDC-funded Emerging Viral Diseases Expert Laboratory Network (EVD-LabNet).

The EQA focused mainly on *Aedes*-borne viruses, i.e., CHIKV (alphavirus), DENV, ZIKV, and YFV (orthoflaviviruses). Also, Japanese encephalitis virus (JEV), an orthoflavivirus that is spread by mosquito species belonging to the genus *Culex*, was included in the EQA panel. Epidemic rise of JEV in regions with visitors returning to Europe or the return to Europe after extensive visits to rural areas with endemic viral presence incidentally gives rise to imported cases (38, 39). Although these cases are not expected to lead to local transmission as humans are dead-end hosts for JEV, JEV was included in the proficiency panel to assess orthoflavivirus test specificity and to improve JEV diagnostic capacity for health care and surveillance purposes. Here, we report the results of this EQA.

## MATERIALS AND METHODS

### Participants

In October 2023, 65 laboratories of EVD-LabNet were invited to register for this EQA or to forward the invitation to competent laboratories in their respective country.

### Panel composition

The EQA panels were designed for molecular detection of CHIKV, DENV, ZIKV, YFV, and JEV using clinically relevant RNA loads and consisted of 12 lyophilized, individually coded samples: 10 plasma samples spiked with one of nine alphavirus or orthoflavivirus species and two RNA-negative plasma samples. Each panel included two CHIKV RNA-positive samples (ESCA and Asian lineages), two ZIKV RNA-positive samples (Asian and African lineages), and one RNA-positive sample for each of the following orthoflaviviruses: DENV subtypes 1–4, JEV, and YFV (Table 1). The viral strains (Table 1) used in these panels were provided by the European Virus Archive-GLOBAL (EVAg) and can be accessed at https://www.european-virus-archive.com/.

**TABLE 1:** Composition of the proficiency panels in the EQA of molecular detection for CHIKV, DENV, ZIKV, YFV, and JEV in EVD-LabNet laboratories, 2023

EQA sample content (virus/lineage)		Virus strain	RNA copies/µL	EVA^*a*^ Ref
Chikungunya virus	ECSA^*c*^	UVE/CHIKV/2006/RE/LR2006_OPY1	1.18E+06	001v-EVA83
Chikungunya virus	Asian	H20235/STMARTIN/2013	7.81E+04	001v-EVA1540
Zika virus	Asian	H/PF/2013	1.73E+04	001v-EVA1545
Zika virus	African	MR766	4.83E+04	001v-EVA143
Dengue virus-1	Genotype I	UVE/DENV-1/2017/TH/7011	1.33E+04	001V-03001
Dengue virus-2	Cosmopolitan Genotype	UVE/DENV-2/2018/RE/47099	3.32E+04	001V-02854
Dengue virus-3	Genotype III	UVE/DENV-3/2015/TH/7716	2.32E+04	001V-03356
Dengue virus-4	Genotype IIb	UVE/DENV-4/2014/HT/6169	8.87E+02	001V-03373
Japanese encephalitis virus	Genotype I	UVE/JEV/2009/LA/CNS769	1.10E+04	001V-02217
Yellow fever virus	South America II	UVE/YFV/1999/BO/BOL88_1999	3.17E+04	001v-EVA1460
Negative control sample (plasma) ×2			NA^*b*^	NA

^*a*^ EVA, European Virus Archive.

^*b*^ NA, not applicable.

^*c*^ ECAS, East-Central-South-African.

### Panel preparation and validation

For the preparation of the EQA panels, Vero E6 cells or C6/36 cells were infected with 1 of the 10 different virus strains (Table 1). The virus culture supernatants were heat inactivated at 60°C for 1 hour. Successful inactivation was confirmed by an additional passage on cells and observation of the absence of cytopathic effect and stable RNA levels over 5 days. Qualified non-therapeutic human plasma provided by the French blood bank was spiked with one of the inactivated virus strains to prepare 0.4-mL aliquots that were freeze dried in glass vials and stored at −20°C until shipment.

The viral loads of EQA specimens were quantified in reference to in-house or specific synthetic RNA controls. A fragment containing the virus-specific TaqMan-targeted sequence and tagged at the 5′-end with the T7 promoter sequence (5′-TAATACGACT CACTATAGGG-3′) was amplified by reverse transcription-PCR (RT-PCR) using an Access RT-PCR Kit (Promega, Madison, WI). The resulting PCR products were purified and transcribed using a T7 MEGAshortscript Kit (Thermo Fisher Scientific, Waltham, MA). The obtained RNA was purified with MegaClear Purification Kit (Thermo Fisher Scientific, Waltham, MA). RNA concentration was measured using a NanoDrop 1000 (Thermo Fisher Scientific, Waltham, MA) and translated into copy numbers. Real-time RT-PCR was performed using a GoTaq real-time quantitative reverse transcription-PCR (qRT-PCR) Kit (Promega, Madison, WI) on a QuantStudio 12K Flex Real-Time PCR System (Thermo Fisher Scientific, Waltham, MA).

The panel was further validated (blind pre-testing) by laboratory staff at the Central Virology Laboratory, Chaim Sheba Medical Center in Tel-Hashomer, Israel, using in-house, species-specific qRT-PCR assays. The freeze-dried panels were shipped to all participants at ambient temperature.

### EQA result submission and evaluation

We collected the EQA results from an online submission form established on the EU survey platform. Participants were asked to submit methodological information, outcomes of each assay used for their diagnosis, and their final (diagnostic) conclusion for each of the samples in the panel. Submitted results were analyzed using Microsoft Excel version 2302 and R Studio, R version 4.2.0. Maps were created via an ECDC Map Maker tool (EMMa).

## RESULTS

### Participation and testing capacity

**Fig 1:**
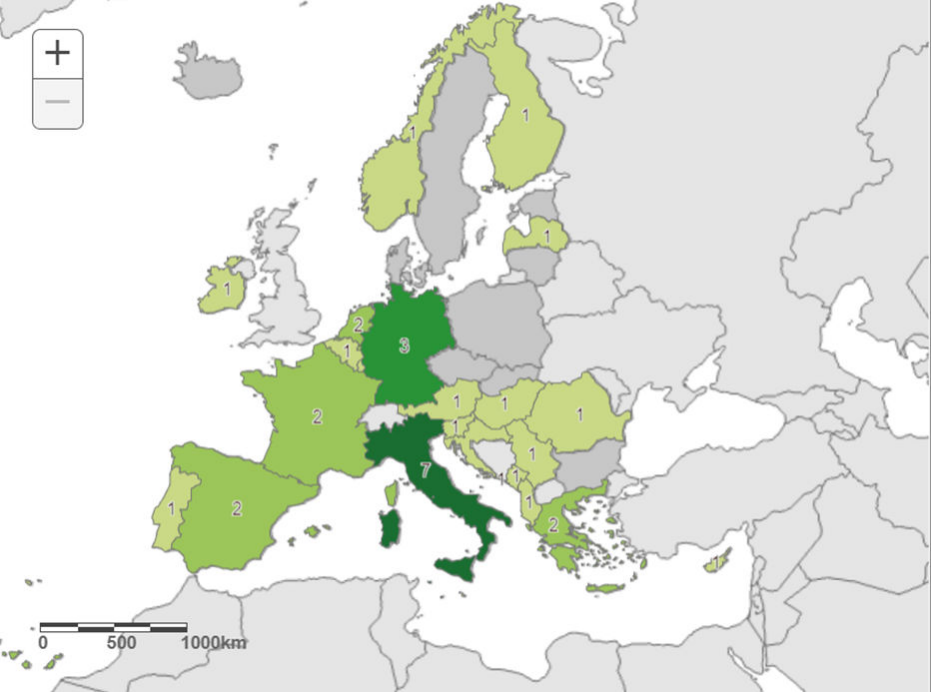
Number of participating laboratories per country. Countries are shaded with green when at least one laboratory in the country completed the mosquito-borne virus EQA panel and reported results. Number in each country is the number of participating laboratories within each country. Darker green means more laboratories in that country participated in the EQA. The number of participating laboratories per country is depicted in the map. The map was created using the ECDC Map Maker tool (EMMa).

In total, 37 laboratories registered to participate and received EQA panels; among the laboratories that registered, one did not submit results. Finally, 36 laboratories from 20 EU/EEA and 4 EU-enlargement countries participated in the EQA (Fig. 1). All 36 laboratories participated with testing for CHIKV, DENV, and ZIKV. Twenty-three laboratories in 13 countries participated with testing for JEV while 30 laboratories in 20 countries participated with testing for YFV (Table 2).

**TABLE 2:** Testing capacity by virus species through species-specific assays (left) and overall (using all diagnostic strategies: virus species-specific, genus-specific RT-PCR assays) by 36 EVD-LabNet laboratories

	No. of labs having species-specific PCR assays	No. of labs having testing capacity (all diagnostic strategies included)
CHIKV	35	36
ZIKV	36	36
DENV	35	36
DENV + subtyping	Not applicable	29
JEV	15	23
YFV	23	30
Pan-alphavirus	Not applicable	7 (2 in combination with sequencing)
Pan-orthoflavivirus	Not applicable	14 (5 in combination with sequencing)

The number of laboratories that used virus species-specific RT-PCR assays for CHIKV, DENV, and ZIKV was very high (ZIKV, 36 of 36; DENV, 35 of 36; and CHIKV, 35 of 36) (Table 2). Less laboratories had both species-specific and subtyping capabilities for DENV (29 of 35), as some assays that are species specific for DENV only read out DENV±, while others read out the DENV subtypes DENV1–4± (Table 2). The number of virus species-specific RT-PCR assays for JEV was 15/23 and that for YFV was 23/30. Regarding genus-specific RT-PCR tests, more laboratories used pan-orthoflavivirus assays (14 of 36) than pan-alphavirus assays (7 of 36). Nine laboratories used high-throughput sequencing (HTS), either as the primary diagnostic test (1 of 36) or in combination with pan-alphavirus or pan-orthoflavivirus assays (8 of 36). Most laboratories (22) used automated extraction, while 14 used manual extraction for nucleic acid preparation.

### Diagnostic conclusions

Considering the final diagnostic conclusion submitted by the participants for each panel entry (including both positive and negative samples), overall, 17 of 36 laboratories (47.2%) identified all EQA samples correctly (Table 3). All the laboratories that could only identify at the genus level called the samples as positive and negative correctly; the laboratories that could identify through a combination of a pan-genus assay and sequencing all identified the panel correctly as well. Incorrect answers were all reported using virus species-specific assays. Six laboratories showed problems of specificity (wrong virus identified in a positive sample) or possible laboratory contamination (false-positive result in a negative control [NC] sample), and three laboratories had problems of sensitivity (at least one false-negative result); no laboratories lacked both sensitivity and specificity.

**TABLE 3:** Final diagnostic outcome reported by all laboratories^*a*^

Lab no.	CHIKV ESCA	CHIKV Asian	ZIKV Asian	ZIKV African	DENV1	DENV2	DENV3	DENV4	JEV	YFV	NC	NC
1	C	C	C	C	C	C	C	C	C	C	C	C
2	C	C	C	C	C	C	C	C	C	C	C	C
3	C	C	C	C	C	C	C	C	C	C	C	I
4	C	C	C	C	C	C	C	C	NA	FN	C	C
5	C	C	C	C	C	C	C	C	NA	NA	C	C
6	C	C	C	C	C	C	C	C	NA	C	C	C
7	C	C	C	C	C	C	C	FN	FN	C	C	C
8	C	C	C	C	C	C	C	C	C	C	C	C
9	C	C	C	C	C	C	C	C	NA/IP	C	C	FP
10	C	C	C	C	C	C	C	C	C	C	C	C
11	C	C	C	C	C	C	C	C	C	C	C	C
12	C	C	C	C	C	C	C	C	NA	NA	C	C
13	C	C	C	C	C	C	C	C	C	C	I	FP
14	C	C	C	C	C	C	C	C	C	C	C	C
15	C	C	C	C	C	C	C	C	C	C	C	C
16	C	C	C	C	C	C	C	C	C	C	C	FP
17	C	C	C	C	C	C	C	C	C	C	C	I
18	C	C	C	C	C	C	C	C	C	C	C	C
19	C	C	C	C	C	C	C	C	C	C	C	C
20	C	C	C	C	C	C	C	C	C	C	C	C
21	C	C	C	C	C	C	C	C	C	C	C	C
22	C	C	C	C	C	C	C	C	C	C	C	C
23	C	C	C	C	C	C	C	C	FN	C	C	C
24	C	C	C	C	C	C	C	C	NA	NA/IP	I	I
25	C	C	C	C	C	C	C	C	C	C	C	C
26	C	C	C	C	C	C	C	C	C	C	C	FP
27	C	C	C	C	C	C	C	C	FN/IP	C	C	C
28	C	C	C	C	C	C	C	C	C	C	C	C
29	C	C	C	C	C	C	C	C	C	C	C	C
30	C	C	C	C	C	C	C	C	NA	C	C	C
31	C	C	C	C	C	C	C	C	NA	NA	C	C
32	C	C	C	C	C	C	C	C	NA	NA	C	C
33	C	C	C	C	C	C	C	C	NA	C	C	C
34	C	C	C	C	C	C	C	C	NA	C	C	C
35	C	C	C	C	C	C	C	C	NA	C	C	C
36	C	C	C	C	C	C	C	C	NA	NA	C	C
**Total correct**	36/36	36/36	36/36	36/36	36/36	36/36	36/36	35/36	20/23	29/30	36/36	32/36
**False**	0/36	0/36	0/36	0/36	0/36	0/36	0/36	1/36	3/23	1/30	0/36	4/36
**Total sensitivity**	100%	100%	100%	100%	100%	100%	100%	97.2%	86.9%	96.6%	NA	NA

^*a*^ Laboratories are anonymized by a unique identifying number. Laboratories submitted final diagnostic results. Results are coded according to capacities indicated by each laboratory. C = correct result based on indicated capacity; NA = test not available, correctly indicated as negative; NA/IP = test not available, incorrectly indicated as positive for other virus species; FN = false negative (test available); FN/IP = false negative (test available), incorrectly indicated as positive for other virus species; FP = false positive; I = inconclusive result; CHIKV = chikungunya virus; DENV = dengue virus; YFV = yellow fever virus; ZIKV = Zika virus; JEV = Japanese encephalitis virus; NC = negative control (no arbovirus RNA).

**Fig 2:**
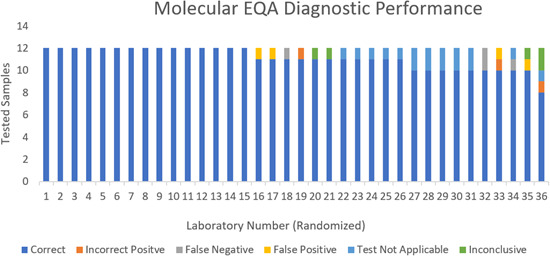
Final diagnostic conclusions provided by laboratories with capacity testing for each positive and negative sample.

The overall performance of individual laboratories for each positive sample, as well as for NCs, is presented in Fig. 2. Correct result rates were 100% for both lineages of CHIKV and both lineages of ZIKV (i.e., all laboratories correctly identified CHIKV RNA-positive and ZIKV RNA-positive samples) (Fig. 2; Table 3). For DENV RNA-positive samples (*n* = 4 per panel and *n* = 144 in total), results were also excellent with only one laboratory having one false-negative result in the DENV-4 RNA-containing sample. Overall, for all DENV samples, 143 of 144 samples were diagnosed correctly. However, one laboratory mis-identified the YFV-RNA panel entry for DENV-RNA, possibly indicating a specificity issue or other misinterpretation issue. Twenty-nine of 36 laboratories were able to correctly sub-type the four DENV-RNA-containing samples. The remaining seven laboratories had no capacity for DENV subtyping. For the JEV-RNA-positive sample in the panel, the primary issue was a lack of capacity in the participant laboratories. Twenty-three of 36 laboratories reported that they have capacity to diagnose JEV samples using molecular diagnostics. At the country level, this corresponded to 13 of 24 participating countries having capacity. Of the 23 laboratories that attempted to diagnose the JEV-positive sample, two laboratories had a false-negative result for the JEV-RNA panel entry while one other laboratory identified the JEV-RNA panel entry as being YFV-RNA positive instead of JEV-RNA positive. For YFV-positive samples, the lack of capacity was also an issue, albeit smaller than JEV. Thirty of 36 laboratories reported that they have the capacity to diagnose YFV samples using molecular diagnostics. At the country level, this corresponded to 20 of 24 participating countries having YFV diagnostic capacity. Only four of 24 countries lacked molecular capability for both YFV and JEV. One laboratory reported a false-negative result for YFV, and two other laboratories reported an incorrect-positive result for YFV in the JEV-RNA panel entry, indicating a specificity problem in the YFV test. The five “inconclusive” results in the NC samples do suggest an interpretation issue for some of the laboratories. Finally, 4 of 36 laboratories reported false-positive results for NC samples. The two NCs were correctly identified as negative by most laboratories (95% of diagnostic conclusions were true negative). Of note, there were four instances (4 of 72) of false positives in the negative panel entries and all false-positive samples were called DENV positive.

### Assay performances by method

Most laboratories used RT-PCR assays to test the proficiency panel. The range of variation in RT-PCR tests was very broad, and numerous laboratories provided information that was not specific enough to identify the exact test used.

Since RT-PCR assays represented the most common diagnostic strategy among participant laboratories (there was one laboratory that reported only based on metagenomic sequencing), we compared outcomes of virus species-specific RT-PCR and pan-alphavirus or pan-orthoflavivirus RT-PCR (regardless of real-time or conventional design). We found that when used as the diagnostic method, pan-orthoflavivirus and pan-alphavirus assays performed perfectly in this EQA. Laboratories that used HTS as a primary or supplemental diagnostic (for example, some laboratories performed pan-alphavirus PCR assays plus sanger sequencing) were also perfect in their diagnosis. Two of the four DENV-RNA false positives in the NCs were from the RealStar Dengue RT-PCR Kit; the other two false positives were each from different molecular assays. The laboratory that misidentified the YFV-RNA panel entry for DENV-RNA used the RealStar Dengue RT-PCR as well. Furthermore, there were two JEV-RNA panel entries that were mislabeled for YFV-RNA. In one instance, a commercial kit was used (VIASURE), and in one instance, an in-house test targeting viral gene NS5A. In both instances, a CT value of >39 was reported.

## DISCUSSION

Thirty-six laboratories in 24 countries (20 EU/EEA, 4 EU-enlargement) participated in this EQA exercise focusing on the molecular detection of three arboviruses (DENV, CHIKV, and ZIKV) (potentially) emerging in Europe in *Ae. albopictus*-infested areas and two arboviruses (YFV, JEV) that are occasionally detected in travelers returning from endemic/epidemic regions worldwide.

Importantly, all laboratories reported the capacity for the three viruses that have proven potential for autochthonous transmission in specific regions in Europe; all participants correctly identified the ZIKV- and CHIKV-RNA-positive panel entries (100% score) while for DENV, only one sample of DENV4-RNA was missed by a laboratory (99.3% score). However, four laboratories identified one of the NC samples as DENV-RNA positive while one laboratory mis-identified the YFV-RNA sample as DENV-RNA positive. At this time (2024), (false) positivity in a suspected autochthonous case in Europe will lead to an extensive local public health response (40).

Molecular detection for CHIKV, DENV, and ZIKV was included in EVD-LabNet proficiency panels in the past. During the ZIKV Public Health Emergency of International Concern, only 40% (20 of 50) of the EVD-LabNet laboratories scored sufficiently, i.e., they had at least one test operational that scored all core samples correctly. Comparing the EQA results in 2016 and 2023 for panel entries with similar RNA loads, we observe an increase in percentage correct results for the ZIKV African lineage from 66% to 100% and for the Asian lineage from 79% to 100% (41).

CHIKV was also included in a 2022 EVD-LabNet proficiency panel targeting molecular detection of alphaviruses. The 2022 CHIKV Asian lineage panel entry with a similar RNA load as in the panel assessed here showed similar results with only one single false-negative result in 2022 versus the 100% score here.

A network EQA in 2003 showed 73%–100% capability to detect DENV1-4; however, only 15 laboratories participated versus 36 laboratories now, representing a significant increase in diagnostic capacity in the EU/EEA. The 2003 EQA showed a profound lack of sensitivity for DENV detection among the participants (42). As sensitivity was not assessed in the current EQA, it is recommended to include a DENV1-4 sensitivity assessment in near-future proficiency tests as part of the work of the soon-to-be-established EURL vector-borne viral diseases laboratory network (43).

For molecular detection of YFV-RNA, 76% of the participants with reported detection capacity reported having a species-specific RT-PCR. The capability among the 30 laboratories was high; only one missed the YFV-RNA-positive sample (a possible sensitivity issue, overall 97% laboratories correct) while another laboratory identified wrongly two other panel entries as YFV-RNA positive (a possible specificity issue). This success rate is a considerable capability improvement in comparison to the previous YFV-dedicated network EQA in 2018, where only 71% of the network laboratories scored a panel entry with South America lineage YFV-RNA with a similar load correct (44).

The capability for molecular detection of JEV RNA was assessed for the first time within EVD-LabNet. The capacity for molecular detection of JEV was the lowest for all five target viruses with 23 of 36 laboratories having the capacity, of which 15 had a JEV-specific RT-PCR test. The success rate was 83% with four laboratories mislabeling the JEV panel entry: 2× it was called negative for arbovirus RNA and 2× it was called as containing YFV-RNA based on very high Ct-values. As the environmental conditions in the EU/EEA are considered suitable for JEV transmission upon introduction of the virus from JEV-endemic regions, laboratory preparedness and response planning for timely and reliable detection in animal reservoirs (birds and pigs) and dead-end hosts like humans and equids are necessary to contain emergence and possible establishment of the virus in bird/pig-mosquito cycles (45) (46). Seeing the importance of reliable and sufficient capacity for JEV detection for potential future JEV risk mitigation, the need for EQA support to the establishment of such capacity is evident. Therefore, besides encouragement of laboratories to implement JEV diagnostics, proficiency testing for molecular detection of JEV is recommended for the future including the range of different JEV genotypes and a sensitivity assessment.

The EQA described here showed once again the high variety in diagnostic tests that are used in EU/EEA laboratories for diagnosis of emerging viral infections and the importance of laboratory-developed tests (LDT) for diagnostic workflows for molecular detection of emerging viruses that are not routinely part of diagnostic portfolios (Table 4). Most laboratories relied on in-house assays with scientific literature as a pivotal source to implement these LDTs. The wide range of LDTs used in this EQA shows that the implementation of LDTs is of high importance for reliable testing in the workflows of the participating laboratories. In 2024, the *in vitro* diagnostic regulation (IVD-R) becomes effective in the EU/EEA (47). Seeing the high reliance of European expert laboratories on LDTs that performed with high reliability in this EQA, the effects of the implementation of the IVD-R on the reliability but also the timeliness of laboratory response operations should be evaluated and assessed very carefully. The observed absence of standardization for arbovirus detection, albeit with great reliability, potentially hampers consistent case finding and reporting for the EU-notifiable diseases caused by DENV, CHIKV, ZIKV, and YFV. To assess whether the molecular capabilities are indeed sufficient for consistent case finding, future EQAs should assess sensitivity aspects of testing more in-depth as RNA loads were quite high in the current proficiency panel and may not represent viral loads seen in clinical settings. Laboratories participating in EQAs are further encouraged to provide more detailed information on the diagnostic tests used to guide putative corrective actions needed and to increase the impact that proficiency testing can have on the quality of testing.

**TABLE 4:** Overview of self-reported diagnostic assays used by 36 laboratories that participated in the EQA for arboviruses^*a*^

Diagnostic assays	# of laboratories using the assay	Performance comments
CHIKV Commercial		
Altona RealStar Chikungunya RT-PCR Kit	8	
Clonit SRL CHIKV	1	
CHIKV LDT		
CHIKV—E1 (48)	5	
CHIKV—nsp1 (49)	3	
CHIKV—non-structural polyprotein (50)	1	
CHIKV—nsp2 (51)	1	
CHIKV—polyprotein (52)	1	
CHIKV—E (53)	1	
CHIKV—3′UTR/NSP1 (54)	1	
CHIKV—unspecified	2	
DENV Commercial		
Altona RealStar Dengue Type RT-PCR Kit	8	False positive (*n* = 2), Incorrect positive (*n* = 1)
Clonit SRL DENV1–4	1	False positive (*n* = 1)
LightMix Reflex Dengue Typing TibMolBiol DENV1–4	1	
Roche LightMix DENV1–4	1	
Sacace Biotechnologies DENV1–4	1	
Viasure CerTest Biotech DENV1–4	1	
DENV LDT		
CDC DENV—1–4 rRT-PCR multiplex assay	4	
DENV1-4—NS5, E, prM x 2 (55)	2	
DENV—3′UTR (56)	2	
DENV1–4—3′UTR (57)	1	
DENV1–4—NS5, E, prM × 2 (58)	1	
DENV1–4—5′UTR-capsid (52)	1	
DENV1–4—E, E/NS1 (59)	1	
DENV1—NS5; DENV2-4-capsid (60)	1	
DENV—unspecified	3	False negative (*n* = 1)
ZIKV Commercial		
Altona RealStar Zika Virus RT-PCR Kit	10	
Clonit SRL ZIKV	1	
Roche LightMix ZIKV	1	
ZIKV LDT		
ZIKV—E (61)	7	
ZIKV—NS1/NS2A (62)	2	
ZIKV—NS5 (63)	1	
ZIKV—3′UTR (51)	1	
ZIKV—NS5A (64)	1	
ZIKV—unspecified	3	
YFV Commercial		
Altona RealStar Yellow Fever Virus RT-PCR Kit	7	
Viasure YFV kit	1	Incorrect positive (*n* = 1)
YFV LDT		
YFV—5′UTR (65)	3	
YFV—5′UTR (66)	3	False negative (*n* = 1)
YFV—5′UTR (67)	1	
YFV—NS1 (68)	1	
YFV—unspecified	6	False positive (*n* = 1)
JEV Commercial		
Liferiver JEV	1	
JEV LDT		
JEV—NS1 (69)	3	
JEV—3′UTR (70)	2	
JEV—NS2A (71)	2	
JEV—5′UTR (67)	1	
JEV—NS5 (72)	1	
JEV—NS2A (73)	1	
JEV—unspecified	3	False negative (*n* = 1)
Multiplex Commercial		
Fast Track Diagnostics Tropical Fever Core (DENV/CHIKV)	2	
Fast Viral Master Mix (custom made)—Life Technologies DENV1 = NS5 gene, DENV2 = C gene, DENV3 = C gene, DENV4 = C gene, pan-DENV = 3′UTR, CHIKV = NSP1 gene, ZIKV = envelope (protein E), YFV = 5NC, JEV = NS5	1	
Roche DENV, ZIKV, CHIKV	1	
Viasure ZIKV, DENV, CHIKV Kit	1	
Illumina viral surveillance panel (sequencing)	1	
Multiplex LDT		
Pan-orthoflavivirus—NS5 (74)	6	
CDC Trioplex rRT-PCR assay (CHIKV, DENV, ZIKV)	5	
Pan-alphavirus—nsP4 (75)	3	
Pan-alphavirus—nsP4 (76)	2	
Pan-alphavirus—nsP4 (77)	2	
Unspecified pan-orthoflavivirus	2	
Pan-orthoflavivirus PCR—NS5 + DENV4 + YFV targets, adapted from reference (72)	1	Incorrect positive (*n* = 1)
Pan-orthoflavivirus—NS1 (78)	1	
Pan-Orthoflavivirus—3′UTR + partial sequencing (79)	1	
Unspecified—target is CHIKV, ZIKV, YFV E1	1	
ZIKV NS5 and detection DENV polyprotein	1	False positive (*n* = 1)
Pan-orthoflavivirus—NS5A (80)	1	
WNV and pan-orthoflavivirus—5′UTR, C, NS5, nsP4 (81)	1	
Pan-alphavirus—unspecified	1	
Other viruses		
High-throughput sequencing	5	
WNV—5′UTR/capsid gene unspecified	1	
MAYV—E2 (82)	1	
TBEV—NovaplexTick-borne Disease Expanded Assay (RUO)	1	
USUV—NS5 (83)	1	
WNV—5′UTR/non-structural protein NS2A (84)	1	
USUV—NS1 (85)	1	
TBEV—5′UTR-capsid (86)	1	
WNV ELITe MGB Kit	1	
USUV—E (87)	1	
WNV—3′UTR-capsid (88)	1	
Altona RealStar WNV RT-PCR Kit	1	
MAYV—unspecified	1	

^*a*^ For the "Performance comments” column, if there is no comment, there were no issues with diagnostic workflows that used that specific diagnostic assay in this EQA. If there is an issue with the performance of diagnostic workflow in which the specific test was used, in the given cell, the issue will be described and the total number of issues will be given (for example, false negative [*n* = 2]). False positive = positive result in negative control; incorrect positive = positive result but incorrect virus species identification. CHIKV = chikungunya virus; DENV = dengue virus; ZIKV = Zika virus; YFV = yellow fever virus; JEV = Japanese encephalitis virus; WNV = West Nile virus; USUV = Usutu virus; TBEV = tick-borne encephalitis virus; MAYV = Mayaro virus; LDT = laboratory-developed tests.

Finally, this EQA was limited to molecular detection of a range of mosquito-borne viruses. While molecular detection of the viral RNAs is a powerful tool in the establishment and confirmation of infection, diagnosis is often based on serology alone as viremia is typically short lived in these infections. Therefore, it is recommended to perform EQAs for detection of virus-specific antibodies in the future. However, the performance of such EQA might be hampered by the availability of sufficient, well-defined serum samples for the rare viruses like JEV and YFV and by legal constraints for distribution of such clinical materials.
